# Marfan's cardiomyopathy is associated with aortic annular and root dilatation in the absence of significant valvular regurgitation

**DOI:** 10.1186/1532-429X-11-S1-O56

**Published:** 2009-01-28

**Authors:** Joyce Wong, Francisco Alpendurada, Elizabeth Burman, Raad Mohiaddin

**Affiliations:** Royal Brompton Hospital, London, UK

**Keywords:** Left Ventricular Ejection Fraction, Cardiovascular Magnetic Resonance, Marfan Syndrome, Aortic Dimension, Marfan Patient

## Introduction

Marfan syndrome is the commonest inherited disorder of connective tissue affecting multiple organ systems, caused by heterozygous mutations in the gene (*FBN1*) that encodes the extracellular matrix protein fibrillin-1. Limited evidence is available that Marfan syndrome is associated with a primary cardiomyopathy. Cardiovascular Magnetic Resonance (CMR) plays an important role in the identification and evaluation of cardiovascular disease in this population, the major source of morbidity and mortality. CMR was used to assess the prevalence and predictors of primary cardiomyopathy, in a Marfan population without evidence of significant valvular regurgitation or aortic disease.

## Methods

120 Marfan patients were consecutively referred to our centre for cardiovascular assessment with CMR between January 2003 and June 2007 (diagnosis based on Ghent criteria). Our study population consisted of 66 of these patients who had no significant valvular regurgitation, and no previous aortic or cardiac disease warranting surgery. This included 38 males (57.8%) and 28 females (42.1%), with a mean age of 33,2 years. Thoracic aortic dimensions were evaluated. Left ventricular (LV) volumes, ejection fraction, and mass were evaluated using semi-automated analysis software (CMRtools, Cardiovascular Imaging Solutions, London, UK). The volumes and mass were then indexed to the body surface area. Aortic (AV) and mitral valves (MV) anatomy and function were assessed, and thoracic musculoskeletal deformities identified. The final values were compared with previously published normal values with values being considered abnormal if they were outside the 95% confidence interval. We similarly compared our population to an age and sex matched cohort of normal individuals when assessing aortic dimensions.

## Results

A significant proportion of this population demonstrated reductions in LV ejection fraction (27%). LV indexed volumes were also significantly different. Annular and aortic root dimensions were increased in the population with reduced LV ejection fraction when compared with population with normal ejection fraction (r of 0.33 and 0.26 respectively, p < 0.05 for both values, Pearson's coefficient). There was no significant difference in global aortopathy indices or incidence of musculoskeletal deformities between the two groups.

## Conclusion

We confirm that Marfan syndrome is associated with a primary cardiomyopathy in a population without significant valvular disease. Moreover, annular and root dimensions are increased in patients with LV dysfunction when compared with patients with preserved LV systolic function, suggesting that aortopathy and cardiomyopathy could be related in this multi-systemic disorder. Recent advances in the molecular pathogenesis of Marfan syndrome suggest that modulation of abnormal fibrillin-1-TGF beta signalling by angiotensin-II receptor blockade in Marfan patients may reverse aortopathy. It remains to be determined whether angiotensin-II receptor blockade may reverse aortopathy and associated cardiomyopathy.

